# MED42/480: Quick Time Virtual Reality (QTVR) Visualization of 2D and 3D Structures in Medical Education

**DOI:** 10.2196/jmir.1.suppl1.e76

**Published:** 1999-09-19

**Authors:** J Dørup, M Schacht Hansen, RB Trelease

**Affiliations:** ^1^University of Aarhus, AarhusDenmark

**Keywords:** Medical Education, Quicktime Virtual Reality, Anatomy, Computer Assisted Learning

## Abstract

**Introduction:**

QTVR is a movie format, developed by Apple, that enables users to work interactively with 2D and 3D objects in a variety of ways that enhance visualization as compared to still images or linear movie files. This standard has been available for some years but the potentials of its use in medical education has not yet been fully exploited.

**Methods:**

Like linear quick time movies, QTVR movies can be readily viewed with an Internet browser (Netscape or Internet Explorer versions 3 or higher) when quick time viewer (available free from Apple) has been installed. Thus the format is well suited for integration into educational web pages. QTVR movies are non-linear movie files that can be manoeuvred by users to:

1. turn 3D objects
2. zoom in and out of 2D or 3D objects
3. pan/scroll objects
4. activate new QTVR movie files by clicking on hot spots

Each frame in the QTVR movie may itself be a movie. QTVR movies are created with the program QTVR authoring studio on Macintosh computers but resulting files are cross-platform compatible and can be distributed on the Internet or on a CD.

**Results:**

The versatility of the tool enables a large variety of different applications to be made (see:http://www.intermed.dk/qtvr for examples and links to other QTVR resources):

1. Gross-anatomical visualization of 3D objects can be created by recording a series of images around a real 3D object suspended in a rotating rag.
2. Panoramic movies can be created by rotating the camera and subsequently stitching images in QTVR authoring studio.
3. A virtual light- or electron microscope can be created by using a 2D image digitized at high resolution.
4. Serial sections of i.e. Magnetic Resonance images or CT images can be manoeuvred by the user to reveal anatomical structures for study.

In one example we have placed a sequence of serial MR movies each representing the cardiac cycle. When this movie is moved using the mouse, another plane of the heart is shown, revealing a very dynamic representation of the entire heart.

**Discussion:**

We plan to further exploit this tool and expect it to be very effective in enhancing interactive learning in topics such as anatomy, cell biology, histology, radiology and cardiology.

